# Short and Long-Term Clinical Outcomes in Octogenarian Patients With Non-ST-Elevation Myocardial Infarction: A Comparative Analysis of Revascularization Strategies Versus Medical Management

**DOI:** 10.7759/cureus.51430

**Published:** 2024-01-01

**Authors:** Fawaz S Baalaraj, Mohammed E Almalki, Mansour M Almalki, Dyaa E Habeeb, Shahad T Abdulrahman, Murouj Almaghrabi, Saad F Alqahtani, Mutaz F Munshi, Ismail Alghamdi, Abdalla Alzoobiy, Ahmed Taha, Mamdouh Ismail, Abdullah Ghabashi, Mohammad O Otain, Saleh M Khouj

**Affiliations:** 1 Medicine, Umm Al-Qura University, Makkah, SAU; 2 Internal Medicine, Umm Al-Qura University, Makkah, SAU; 3 Preventive Medicine, Umm Al-Qura University, Makkah, SAU; 4 Cardiology, Umm Al-Qura University, Makkah, SAU; 5 Cardiology, King Abdullah Medical City, Makkah, SAU

**Keywords:** mortality, cabg, percutaneous coronary intervention, nstemi, revascularization

## Abstract

Introduction: As the primary cause of morbidity and mortality among older individuals, cardiovascular disease remains a major concern. Choosing between revascularization and medical management of elderly patients remains controversial. This study aims to evaluate the clinical implications of these treatment approaches in the context of non-ST-elevation myocardial infarction (NSTEMI) in octogenarian patients.

Methods: This observational cohort study involved 41 octogenarian patients who were diagnosed with NSTEMI from 2019 to 2021 and were managed by revascularization (with either percutaneous coronary intervention, coronary artery bypass graft surgery, or both) or conservative medical therapy. All NSTEMI patients were diagnosed based on symptoms, electrocardiographic changes, and cardiac biomarkers. The study compared the short- and long-term outcomes of 13 patients in the revascularization group and 28 in the medical therapy group.

Results: Overall, the mean patient age was 84.63 years. Eighteen patients were men (43.9%), and 23 were women (56.1%). The most prevalent disease among the sample was hypertension (34 patients, 82.9%), followed by diabetes mellitus (27 patients, 65.9%) and prior ischemic heart disease (21 patients, 51.2%). Almost all patients in the revascularization-treated group developed complications after the procedure (84.6%), while 46.4% of the patients in the medication-only group developed a complication later on. The revascularization-treated group showed higher mortality rates in both the short- and long-term (23.1% and 38.5%, respectively) compared to the medication-only group, which showed better survival rates numerically in both the short- and long-term (14.3% and 32.1%, respectively). This was not statistically significant.

Conclusion: Revascularization treatment in elderly patients with NSTEMI was associated with a higher risk of complications and a higher mortality rate compared with conservative medical management. Patients managed with only medications had a better survival rate in both the short- and long-term.

## Introduction

As the leading cause of morbidity and mortality among the elderly population, cardiovascular disease remains a significant concern [1]. With advancements in health care services, the aging population is experiencing an increase in life expectancy, which emphasizes the need for a comprehensive understanding of cardiovascular diseases and effective strategies to address the health challenges they cause [1]. However, regarding treatment options, the choice between revascularization and conservative medical management in elderly patients remains controversial [2,3], necessitating further research. While revascularization has improved clinical outcomes in patients with acute coronary syndrome, its applicability and effectiveness in the elderly population are not yet well established [2,4].

Physicians often opt for a conservative approach when contemplating revascularization procedures for their octogenarian patients, acknowledging the heightened risk of complications associated with revascularization and the lack of supporting evidence for its efficacy in this population [2,5]. This knowledge gap underscores the importance of the present study comparing the effectiveness of revascularization with that of medical management in elderly patients, particularly those with non-ST-elevation myocardial infarction (NSTEMI).

This study also explores long-term outcomes, addressing a gap in the existing literature by examining the effects of these interventions beyond short-term follow-up. Our study aims to provide a comprehensive evaluation of the clinical implications of these treatment approaches in the context of NSTEMI in older adults. The findings from this study will contribute to clinical decision-making and the ongoing discussion surrounding the management of cardiovascular diseases in octogenarians.

## Materials and methods

Study population

This study was designed as a retrospective cohort. It involved all octogenarian patients (defined by the Britannica Dictionary as the age group between 80 and 89 years) who suffered from NSTEMI at King Abdullah Medical City (KAMC) in Makkah, Saudi Arabia, between 2017 and 2021. All patients were included either by direct admission via the emergency department or were referred from the cardiology and medical departments. The authors excluded patients who died within the hospitalization period, as follow-up could not be obtained.

The diagnosis of NSTEMI was made based on the presented symptoms, electrocardiographic changes (ST segment depression or T-wave abnormalities), and cardiac biomarkers. A responsible cardiologist made the treatment strategy decision between revascularization and medical management. All percutaneous coronary interventions (PCIs) were performed at the same center of KAMC.

Ethical consideration

We gained ethical approval from the Institutional Review Board at KAMC, registered at the National Biomedical Ethics Committee at King Abdulaziz City for Science and Technology, on 14-07-1433 (Registration no.: H-02-K-001). Then, data were collected from patients’ electronic files onto electronic data collection forms without nominative information. Patients were identified by serial code and initials linked to their names and MRN in a separate identification log sheet kept in a safe, locked location. Two different authors performed data entry. After verification, data were transferred directly to the statistical database.

Data collection

The collected data included demographic information, history of chronic illnesses, home medications, hospital treatment plan, other clinical diagnoses, laboratory data, final clinical outcome, and complications during the procedure.

Follow-up and end-points

All patients who were diagnosed and managed in KAMC during the study period were followed for a total of three years. The end-points were obtained on two occasions, of 1 year and 3 years. The measured outcomes during the follow-up time included the development of complications (e.g., nonfatal myocardial infarction, stroke, heart failure requiring hospitalization, etc.) as well as cardiovascular disease-related death and all-cause mortality.

Statistical analysis

IBM Statistical Product and Service Solutions (SPSS) (Version 26) was used. Numerical data are presented as mean±SD. For categorical variables, frequencies and percentages are used. Comparisons between groups were made using the Student’s t-test. The chi-square test was used for categorical values. Survival analysis using the Kaplan-Meier curve was included to compare the survival rates between the medications group and the revascularization group in terms of cardiovascular death and all-cause mortality. A 95% confidence interval with a significance level of 5% was used for testing differences between variables.

## Results

A total of 41 patients were included in the study. Table 1 shows the characteristics of the included patients. The mean patient age was 84.63 (± 4.38) years. Eighteen were men (43.9%), and 23 were women (56.1%). The mean body mass index was 27.15 (± 4.43) kg/m2. Mean heart rate was 76.30 (± 13.83) beats per minute, and mean blood pressure was 129.69 (± 27.19)/67.81 (± 17.95) mmHg. Regarding past medical history, the most prevalent disease was hypertension (34 patients, 82.9%), followed by diabetes mellitus (27 patients, 65.9%) and prior ischemic heart disease (21 patients, 51.2%). Additional data are presented in Table 1. Concerning management strategy, only 13 patients (31.7%) underwent revascularization: nine (22.0%) underwent percutaneous coronary intervention (PCI) alone, while four patients (9.8%) underwent PCI plus coronary artery bypass graft (CABG) surgery. Radial access was used in performing the PCI in all but one patient for whom femoral access was used. All remaining patients (28, 68.3%) were managed with medical therapy exclusively. As shown in Table 1, medical treatments used at the presentation included aspirin, which was used in the case of 39 of the patients (95.1%); P2Y12 inhibitors, given to 26 patients (63.4%); and inotropes, given to five patients (12.2%). Table 1 also shows medications used after discharge, which included aspirin, given to 39 patients (95.1%); P2Y12 inhibitors, given to 32 patients (78.0%); beta-blockers, given to 29 patients (70.7%); statins, given to 31 patients (75.6%); and angiotensin-converting enzyme inhibitors/angiotensin-receptor blockers, given to 16 patients (39.0%). Additional data, including laboratory investigations, are also demonstrated in Table 1.

**Table 1:** Patient Characteristics and Management Abbreviations: BMI, body mass index; PAD, peripheral artery disease; PCI, percutaneous coronary intervention; CABG, coronary artery bypass graft; ACE/ARB, angiotensin-converting enzyme inhibitor/angiotensin receptor blocker; LVEF, left ventricular ejection fraction.

Studied variables		No. (%) or Mean (±SD)
Age (years)		84.63 (4.3804)
Sex	Male	18 (43.9)
	Female	23 (56.1)
BMI		27.15 (4.431912)
Heart rate		76.30 (13.8351)
Systolic blood pressure		129.69 (27.1967)
Diastolic blood pressure		67.81 (17.95888)
Past medical history		
Hypertension	No	7 (17.1)
	Yes	34 (82.9)
Diabetes mellitus	No	14 (34.1)
	Yes	27 (65.9)
Dyslipidemia	No	36 (87.8)
	Yes	5 (12.2)
Prior ischemic heart disease	No	20 (48.8)
	Yes	21 (51.2)
Anemia	No	35 (85.4)
	Yes	6 (14.6)
Smoking	No	35 (85.4)
	Yes	6 (14.6)
Prior PAD	No	40 (97.6)
	Yes	1 (2.4)
Revascularization	No	28 (68.3)
	Yes	13 (31.7)
Mode of revascularization	PCI	9 (22.0)
	PCI + CABG	4 (9.8)
Access site for PCI	Femoral	1 (2.4)
	Radial	12 (29.3)
Medical treatment at presentation		
Aspirin	No	2 (4.9)
	Yes	39 (95.1)
P2Y_12_ inhibitor (clopidogrel, ticagrelor)	No	15 (36.6)
	Yes	26 (63.4)
Inotropes (dopamine, dobutamine, norepinephrine)	No	36 (87.8)
	Yes	5 (12.2)
Medications after discharge		
Aspirin	No	2 (4.9)
	Yes	39 (95.1)
P2Y_12_ inhibitor	No	9 (22.0)
	Yes	32 (78.0)
Beta blocker	No	12 (29.3)
	Yes	29 (70.7)
Statin	No	10 (24.4)
	Yes	31 (75.6)
ACE/ARB	No	25 (61.0)
	Yes	16 (39.0)
Creatinine (mg/dL)		1.28 (.567576)
LVEF (%)		43.13 (13.009058)
Troponin-I (ng/dL)		4.89 (9.982463)
Hemoglobin (g/dL)		12.00 (2.109147)

Table 2 compares the two groups regarding various factors, including demographics, clinical history, and medication use. Notably, no significant associations were found between these factors and the type of management (P > 0.05). All-cause mortality also showed no association with the type of management (P > 0.05).

**Table 2:** Comparison Between Patients Who Underwent Revascularization and Those Who Received Only Medications Abbreviations: BMI, body mass index; PAD, peripheral artery disease; ACE/ARB, angiotensin-converting enzyme inhibitor/angiotensin receptor blocker; LVEF, left ventricular ejection fraction; MI, myocardial infarction; HFRH, heart failure requiring hospitalization; CV, cardiovascular.

Studied variables		Medication only	Revascularization	
		No. (%) or Mean (±SD)	No. (%) or Mean (±SD)	P value
Age		86.07 (4.47)	81.53 (1.94)	<0.000
Sex	Male	17 (73.9)	6 (26.1)	0.382
	Female	11 (61.1)	7 (38.9)	
BMI		26.70 (4.19)	28.13 (4.93)	0.342
Heart rate		76.82 (12.19)	75.19 (17.35)	0.730
Systolic blood pressure		134.25 (28.44)	119.88 (22.19)	0.117
Diastolic blood pressure		68.74 (18.13)	65.80 (18.13)	0.632
Past medical history				
Hypertension	No	5 (71.4)	2 (28.6)	0.845
	Yes	23 (67.6)	11 (32.4)	
Diabetes mellitus	No	11 (78.6)	3 (21.4)	0.308
	Yes	17 (63.0)	10 (37.0)	
Dyslipidemia	No	26 (72.2)	10 (27.8)	0.147
	Yes	2 (40.0)	2 (60.0)	
Prior ischemic heart disease	No	14 (70.0)	6 (30.0)	0.819
	Yes	14 (66.7)	7 (33.3)	
Anemia	No	26 (74.3)	9 (25.7)	0.046
	Yes	2 (33.3)	4 (66.7)	
Smoking	No	24 (68.6)	11 (31.4)	0.926
	Yes	4 (66.7)	2 (33.3)	
Prior PAD	No	27 (67.5)	13 (32.5)	0.409
	Yes	1 (100)	0 (0)	
Medical treatment at presentation				
Aspirin	No	1 (50)	1 (50)	0.569
	Yes	27 (69.2)	12 (30.8)	
P2Y_12_ inhibitor (clopidogrel, ticagrelor)	No	11 (73.3)	4 (26.7)	0.598
	Yes	17 (65.4)	9 (34.6)	
Inotropes (dopamine, dobutamine, norepinephrine)	No	25 (69.4)	11 (30.6)	0.671
	Yes	3 (60)	2 (40)	
Medications after discharge				
Aspirin	No	2 (100)	0 (0)	0.323
	Yes	26 (66 .7)	13 (33.3)	
P2Y_12_ inhibitor	No	5 (55.6)	4 (44.4)	0.353
	Yes	23 (71.9)	9 (28.1)	
Beta blocker	No	11 (91.7)	1 (8.3)	0.039
	Yes	17 (58.6)	12 (41.4)	
Statin	No	6 (60)	4 (40)	0.517
	Yes	22 (71.0)	9 (29.0)	
ACE/ARB	No	19 (76.0)	6 (24.0)	0.185
	Yes	9 (56.3)	7 (43.8)	
Creatinine (mg/dL)		1.32 (.56)	1.19 (.59)	0.523
LVEF (%)		43.58 (12.22)	42.14 (15.04)	0.746
Troponin-I (ng/dL)		4.76 (11.08)	5.16 (7.44)	0.906
Hemoglobin (g/dL)		11.87 (1.71)	12.27 (2.84)	0.641
Nonfatal MI	No	25 (69.4)	11 (30.6)	0.671
	Yes	3 (60)	2 (40)	
Stroke	No	27 (67.5)	13 (32.5)	0.490
	Yes	1 (100)	0 (0)	
HFRH	No	25 (69.4)	11 (30.6)	0.671
	Yes	3 (60)	2 (40)	
CV death	No	24 (72.7)	9 (27.3)	0.215
	Yes	4 (50)	4 (50)	
All-cause mortality	No	19 (70.4)	8 (29.6)	0.691
	Yes	9 (64.3)	5 (35.7)	

This study thoroughly assessed the clinical significance and effectiveness of revascularization versus medical therapy in octogenarians with NSTEMI, aiming to compare favorable outcomes between the two groups. The results revealed that a large proportion of patients in the revascularization-treated group (84.6%) developed complications, either due to the nature of their disease or related to the procedure. In the medication-only group, 46.4% of the patients experienced complications, but later (Table 3). Age, anemia, and use of beta-blockers after discharge showed significant association with the type of management (P < 0.000, P < 0.046, P < 0.039, respectively).

**Table 3:** Summary of complications and short- and long-term outcomes among the included patients (n=41) Abbreviations: MI: myocardial infarction; HFRH: heart failure requiring hospitalization; CV death: cardiovascular diseases-related death

Complications	Revascularization group (n=13)	Medical therapy group (n=28)
	n (%)	n (%)
Total	11 (84.6%)	13 (46.4%)
Nonfatal MI	2 (15.4%)	3 (10.7%)
Stroke	0 (0.0%)	1 (3.6%)
HFRH	2 (15.4%)	3 (10.7%)
CV death	4 (30.8%)	4 (14.3%)
Long-term outcomes		
3-Year all-cause mortality	5 (38.5%)	9 (32.1%)
Short-term outcomes		
1-Year CV death	3 (23.1%)	1 (3.6%)
1-Year all-cause mortality	3 (23.1%)	4 (14.3%)

**Figure 1:**
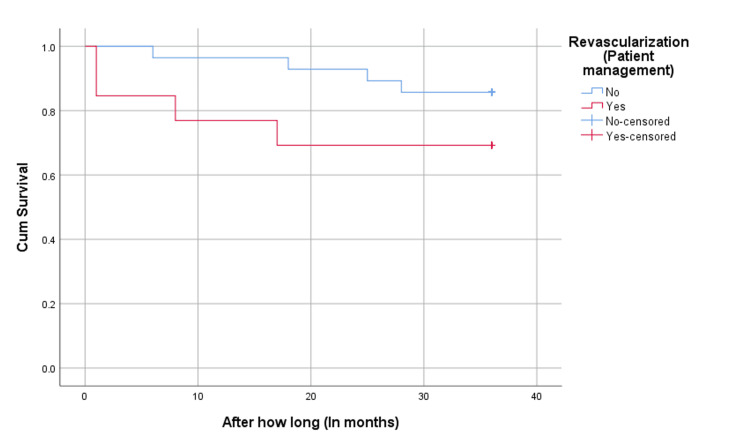
CV death survival analysis *Log-Rank test p-value=0.166

**Figure 2:**
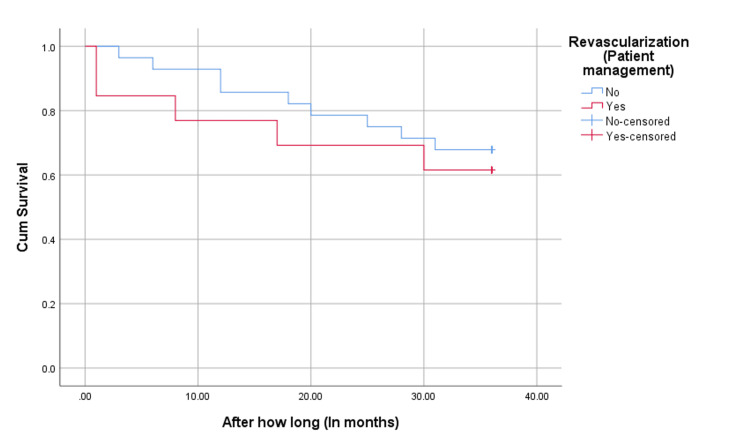
All-cause mortality survival analysis *Log-Rank Test P-Value=0.592

As shown in Figures 1-2, we compared the survival rate between the revascularization and medical therapy groups regarding cardiovascular death and all-cause mortality. The medical therapy group exhibited slightly better survival rates for both cardiovascular death and all-cause mortality, though the differences were not statistically significant (P>0.05).

## Discussion

Our results demonstrate that the revascularization-treated group had a higher mortality rate than the medication-only group, which exhibited better survival in both the short- and long-term. Elderly patients are known to be at higher risk for multiple health conditions and have a greater extent of atherosclerotic disease, making revascularization technically more challenging. However, recent evidence from Germany and Sweden has suggested that PCI in the older population leads to favorable outcomes [6,7]. A previous systematic review and meta-analysis published by McKellar et al. reviewed 32 studies to investigate the outcomes of PCI among the elderly. They concluded that PCI had acceptable short- and long-term outcomes among octogenarians; however, most reviewed studies had low-level evidence [8].

Our results are inconsistent with previous findings, possibly due to several reasons. First, there is the nature of the population. Even without genetic changes, the risk of cardiovascular disease, in general, can be altered by changes in the environment resulting from migration to different geographic locations, modifications in lifestyle choices, and shifts in social policies and cultural practices [9]. Our review of the literature showed no previous studies conducted among the Saudi population on the issue of treatment strategy. Therefore, we suggest conducting local investigations to gain a deeper understanding of the society, which will aid in transitioning from standardized to customized management tailored to the community. Second, methodological aspects may have contributed to the present results, such as the limited number of participants in the study and the difference in the number of patients in the two groups. Third, some of our patients in the revascularization-treated group received further treatment in the form of CABG, which may have contributed to the higher mortality rates, as suggested by previous studies [10-12]. Moreover, a study published by Santos et al. showed a mortality rate of 8.7% among 1,628 patients who underwent CABG and identified the following risk factors: age over 65 years, being on dialysis, having type I neurologic dysfunction, use of intra-aortic balloon pump, serum creatinine level on admission and peak values over 0.4 mg/dL, cardiopulmonary bypass time of more than 115 minutes, and time window between hospital admission to the surgical procedure [13].

Our three-year survival analysis for all-cause mortality revealed a higher mortality rate for the revascularization-treated group than for the medication-only group (38.5% vs 32.1%, respectively) (Table 3). However, the difference was not statistically significant. These findings align with a meta-analysis of randomized controlled trials involving patients with all forms of coronary artery disease. A sensitivity analysis of the longer-term follow-up trials revealed a non-significant reduction in mortality rate in the revascularization-treated group among patients with myocardial infarction. In addition, revascularization did not impact three-year all-cause mortality in patients with stable coronary artery disease [14].

Several factors influence the decision to proceed with revascularization in elderly patients. Comorbid illnesses, clinical frailty, and the patient’s functional status must be considered. Comorbid illnesses include but are not limited to, arthritis and cerebral, renal, and pulmonary diseases [15]. Reports show patients with a lower risk profile were more likely to be selected for revascularization management [16,17]. However, we can still not determine the specific factors influencing the decision to opt for PCI in managing older adult patients. Nevertheless, a previous cohort study confirmed that younger age, male gender, lower heart rate, lower systolic blood pressure, and no history of hypertension or stroke were determinants for selecting PCI for octogenarian patients [6].

Strengths and limitations

This study provided an extraordinary opinion regarding the decision to choose revascularization treatment in the case of octogenarian patients in the Saudi community. The study encourages and lays the groundwork for future randomized trials to be conducted locally. Despite all efforts, the study has several limitations. First, confounding factors from unmeasured variables could not be ruled out completely. Second, the limited sample size and different number of patients in each group may have remarkably affected our findings. This limitation depended on available data on patients who presented to our center between 2017 and 2021, over which we had no control. Lastly, there is the possibility of bias resulting from the study's retrospective nature, as our data were obtained from patient files. Missed documentation and recall bias are inherent limitations that cannot be avoided in this type of study.

## Conclusions

Our study revealed that numerous patients in the revascularization-treated group developed complications after the procedure, while fewer complications were observed among the patients treated with only medication. The study also demonstrated that the revascularization-treated group had a higher mortality rate compared with the medical-therapy group, which showed better survival in both the short- and long-term.

However, these numbers were statistically non-significant. We suggest conducting additional local investigations involving multiple centers, larger numbers of patients, and randomized trials to gain a deeper understanding of our society in Saudi Arabia, which will aid in transitioning from standardized to customized management tailored to the community.
